# Supplementary material for: Prevention of Post-Hepatectomy Liver Failure in Cirrhotic Patients Undergoing Minimally Invasive Liver Surgery for HCC: Has the Round Ligament to Be Preserved?
Source: Cancers (Basel). 2024 Jan 15;16(2):364. doi: 10.3390/cancers16020364 (PMC10814940; doi:10.3390/cancers16020364)
Supplement: Supplementary file 1 [file cancers-16-00364-s001.zip › cancers-2688398-supplementary.pdf]

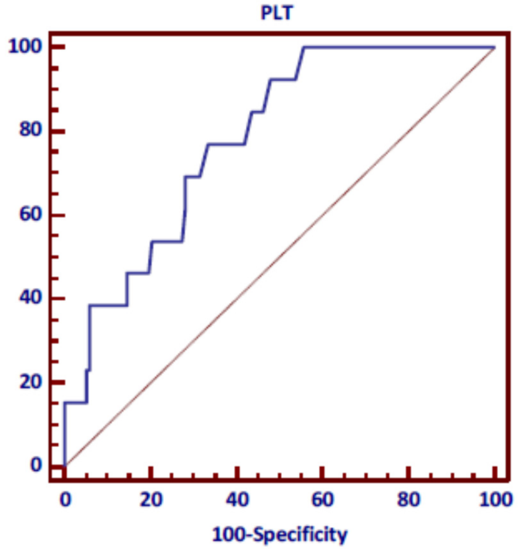

Figure S1. ROC curve analysis of the platelets (PLT) count [< 92 x 103/μL (AUC = 0.781, p = <0.001)] predictive for postoperative ascites.

Table S1. Baseline and procedure characteristics of patients undergoing laparoscopic liver resections with Round Ligament (RL) preservation and RL division excluding patients converted to open surgery.

**Patient characteristics**	**RL divided (n = 31)**	**RL preserved (n = 85)**	p
**Preoperative characteristics**			
Age, years (IQR)	65 (58 - 72)	68 (60 - 75)	0.101
Sex, male (%)	22 (71%)	64 (75.3%)	0.638
BMI, kg/m2 (IQR)	24 (24 - 28)	25 (25 - 29)	0.178
ASA grade			0.308
ASA 1 (%)	0	0	
ASA 2 (%)	18 (58.1%)	58 (68.2%)	
ASA 3 (%)	13 (41.9%)	27 (31.8%)	
ASA 4 (%)	0	0	
Grade of Cirrhosis			0.965
Child Pugh A (%)	26 (83.9%)	71 (83.5%)	
Child Pugh B (%)	5 (16.1%)	14 (16.5%)	
Etiology of Cirrhosis			0.290
Alcohol (%)	1(3.2%)	15 (17.6%)	
HCV (%)	13 (41.9%)	34 (40%)	
HBV (%)	5 (16.1%)	11 (12.9%)	
Metabolic (%)	5 (16.1%)	14 (16.5%)	
Other (%)	7 (22.6%)	11 (12.9%)	
ICG-R15 (IQR)	15.0 (12 – 17)	15.5 (13 -19)	0.780
Platelets count x103/μL (IQR)	104 (86 - 119)	107 (90 - 120)	0.390
Previous abdominal surgery (%)	18 (58.1%)	38 (44.7%)	0.203
Previous liver surgery (%)	7 (22.6%)	15 (17.6%)	0.549
Number of nodules (IQR)	1 (1 – 1)	1 (1 – 1)	0.976
Max. nodule size, mm (IQR)	30 (17 – 37)	25 (14 – 33)	0.314
**Intraoperative Characteristics**			
Type of Resection			0.180
Non-anatomic resection (%)	10 (32.3%)	42 (49.4%)	
Segmentectomy (%)	14 (45.2%)	34 (40.0%)	
Left lateral sectionectomy (%)	6 (19.4%)	5 (5.9%)	
Right posterior sectionectomy (%)	1 (3.2%)	4 (4.7%)	

Pringle manoeuvre (%)	26 (83.9%)	73 (85.9%)	0.786
Duration of Pringle man., min (IQR)	36 (17 - 65)	34 (15 - 40)	0.969
Operative time, min (IQR)	315 (216 – 380)	264 (195 – 300)	0.091
Blood loss, cc (IQR)	100 (30 – 200)	100 (20 – 170)	0.868

Abbreviations: ASA, American Society of Anesthesiology; BMI, body mass index; IQR, inter quartile range, ICG-R15, Indocyanine green – retention rate at 15 min; HCV, hepatitis c virus; HBV, hepatitis B virus.

Table S2. Postoperative outcomes of patients undergoing laparoscopic liver resections with Round Ligament (RL) preservation and RL division excluding patients converted to open surgery.

Postoperative Outcomes	RL divided (n = 31)	RL preserved (n = 85)	*p*
Postoperative complication-30 days¥ (%)	14 (45.2%)	24 (28.2%)	0.086
Severe, grade 3-5 (%)	4 (12.9%)	10 (11.8%)	0.868
Postoperative complication-90 days¥ (%)	14 (45.2%)	26 (30.6%)	0.144
Post Hepatectomy Liver Failure* (%)	9 (29.0%)	8 (9.4%)	0.008
Severe, grade B-C (%)	6 (19.4 %)	6 (7.1 %)	0.049
Ascites (%)	6 (19.4%)	5 (5.9%)	0.028
30-days readmission (%)	1 (3.2%)	5 (5.9%)	0.568
ICU Stay, days (IQR)	1 (0 – 1)	1 (0 – 1)	0.593
Hospital stay, days (IQR)	5 (4 – 7)	5 (4 – 5)	0.596

IQR = inter quartile range, * ISGLS definition (International study group for liver surgery), ¥ Clavien-Dindo Classification.

Table S3. Uni and Multivariate analysis of risk factors associated with the development of postoperative ascites in the whole population excluding patients converted to open surgery.

ASCITES	Univariate Analysis		Multivariate Analysis	
Predictive factors	Odds Ratio (95% CI)	p	Odds Ratio (95% CI)	*p*
**Preoperative characteristics**				
Age, years	0.980 (0.928 to 1.035)	0.467		
Sex, male	0.375 (0.105 to 1.334)	0.130		
BMI, kg/m^2	1.012 (0.870 to 1.176)	0.880		
ASA grade				
ASA 2				
ASA 3	3.654 (1.001 to 13.338)	0.050	2.500 (0.624 to 10.011)	0.196
Grade of Cirrhosis				
Child Pugh A				
Child Pugh B	3.429 (1.004 to 13.154)	0.047	1.953 (0.362 to 10.544)	0.437
Etiology of Cirrhosis				
Alcohol				
HCV	0.758 (0.171 to 3.366)	0.716		
HBV	1.001 (0.999 to 1.002)	0.998		
Metabolic	0.241 (0.022 to 2.583)	0.240		
Other	1.000 (0.999 to 1.000)	0.999		
ICG-R15 (IQR)	1.004 (0.881 to 1.090)	0.585		
Platelets count x10^3/μL	0.900 (0.983 to 1.005)	0.045	0.994 (0.981 to 1.088)	0.430
Previous abdominal surgery	0.882 (0.254 to 3.070)	0.844		
Previous liver surgery	0.944 (0.189 to 4.714)	0.944		
Number of nodules	0.223 (0.018 to 2.796)	0.245		
Max. nodule size, mm	1.003 (0.965 to 1.042)	0.890		
**Intraoperative Characteristics**				
Round Ligament divided	3.840 (1.080 to 13.659)	0.038	4.054 (1.016 to 16.181)	0.047
Type of Resection				
Non-anatomic resection				
Segmentectomy	0.429 (0.104 to 1.763)	0.240		
Left lateral sectionectomy	0.999 (0.998 to 1.002)	0.995		
Right posterior sectionectomy	1.607 (0.156 to 16.543)	0.690		
Pringle manoeuvre	1.001 (0.999 to 1.002)	0.998		
Duration of Pringle man., min	0.997 (0.984 to 1.011)	0.698		
Operative time, min	1.001 (0.995 to 1.007)	0.705		

Blood loss, cc	1.001 (0.099 to 1.002)	0.358

Abbreviations: ASA, American Society of Anesthesiology; BMI, body mass index; IQR, inter quartile range, ICG-R15, Indocyanine green – retention rate at 15 min; HCV, hepatitis c virus; HBV, hepatitis B virus.
